# Postprandial Reactive Hypoglycaemia: Varying Presentation Patterns on Extended Glucose Tolerance Tests and Possible Therapeutic Approaches

**DOI:** 10.1155/2013/273957

**Published:** 2013-01-10

**Authors:** Kevin Stuart, Annmarie Field, Jessie Raju, Sudarshan Ramachandran

**Affiliations:** ^1^Department of Clinical Biochemistry, Good Hope Hospital, Heart of England NHS Foundation Trust, Rectory Road, Sutton Coldfield, Birmingham B75 7RR, UK; ^2^Senior Diabetes Dietician, Department of Nutrition and Dietetics, Heart of England Hospital, Heart of England NHS Foundation Trust, Bordesley Green, Birmingham B9 5SS, UK

## Abstract

Reactive hypoglycemia is a state characterised by sympathetic or neuroglycopenic symptoms associated with hypoglycaemia in the postprandial state resulting in considerable distress to the patient. It is our practice to carry out either extended glucose tolerance tests (eGTTs) or mixed meal tests in these patients. We describe two patients who experienced hypoglycaemic symptoms early and late during eGTT. The patient who experienced symptoms early, in contrast to the patient who presented with late symptoms, did not possess any characteristics of the metabolic syndrome. Based on clinical symptoms, glucose, insulin, and free fatty acid (FFA) levels, we speculate on possible mechanisms that may have accounted for each of their presentation patterns. We then discuss low glycaemic index diet which will be the mainstay of management.

## 1. Introduction

Reactive hypoglycaemia is often considered in patients developing sympathetic or neuroglycopenic symptoms in the postprandial state. Prior to this diagnosis being reached, it is essential that hypoglycaemia must be demonstrated to be associated with the symptoms (http://diabetes.niddk.nih.gov/dm/pubs/hypoglycemia/). It has been suggested that demonstration of hypoglycaemia (glucose lower than 3.9 mmol/L or 70 mg/dL) while symptomatic and alleviation of symptoms following normalisation of glucose levels should replace the extended gluces tolerance test (eGTT). However, this can pose practical problems as patients would need laboratory bloods to be carried out when symptomatic for a reliable diagnosis. In view of this, it has been our practice to carry out either a front-line eGTT or a mixed meal test (in the event of the patient being able to identify a particular meal or food with the symptoms) with insulin and FFA measurements upon the onset of symptoms. Insulin and FFA measurements in our view make it easier to reach a diagnosis of postprandial reactive hypoglycaemia on a physiological basis [1, 2]. There has been considerable debate regarding the level of glucose that defines hypoglycaemia, and this has ranged between 2.2 mmol/L and 4.0 mmol/L [3].

The results of the eGTT carried out in our metabolic investigation unit have shown differing clinical and biochemical patterns requiring an understanding of the relationship between glucose and factors influencing it such as insulin levels, insulin resistance, and counterregulatory measures. We have observed symptomatic hypoglycaemia both early and late during the eGTT. We present a couple of patients demonstrating possible reactive hypoglycaemia at different time points during the eGTT and then speculate on possible causes. This will be followed by possible actions that could be taken, focusing mainly on dietary measures.

## 2. Methods

Patients with possible postprandial reactive hypoglycaemia are often referred to the endocrine or metabolic clinics at Good Hope Hospital and are investigated in the metabolic investigation unit following clinical assessment. The eGTT is not recommended in patients with intercurrent illness, dumping syndrome, or postsurgery due to difficulty in the interpretation of results. It is carried out following a minimum 10 hours of fasting with only water permitted, preceded by 3 days or more of normal diet and activity. Polycal (113 mL which contains 75 g of anhydrous glucose, made up to 200 mL of water) is orally administered, followed by a further 100 mL of water. Blood samples (for glucose, insulin, c-peptide, and free fatty acids) are carried out at 0 minutes and at 30 minutes intervals for 4 hours and also at the point of patient's symptoms. The investigation may require discontinuation if the patient is overtly symptomatic and requires rescue. All the samples are analysed for glucose with insulin, C-peptide, and free fatty acids estimated in the event of low blood sugar (≤3.5 mmol/L), hypoglycaemic symptoms, or retrospectively requested when deemed useful in reaching a diagnosis. Abnormal results are then discussed at a following multidisciplinary team meeting and future management and followup mapped out. The patients are then seen in the out-patient clinics, the results discussed, and possible treatments discussed with appropriate followup.

Glucose was measured using an Enzymatic Hexokinase/G-6-PDH assay on the Abbott Architect C system, the assay CV being 2.2%. FFA was estimated at the Birmingham Children's Hospital using a colourimetric assay on the Olympus 640 analyser. Insulin was measured at the SAS Peptide Hormone Centre, Royal Surrey Hospital, Guilford, using the Mercodia Iso Insulin ELISA Immunoassay.

## 3. Case History

We present 2 patients presenting with hypoglycaemic symptoms during the eGTT at different time points and describe their clinical features, recovery, and biochemical changes. 

### 3.1. Early Hypoglycaemia (Patient A)

Patient A, a 70-year-old woman (weight: 67 kg; BMI: 24.2) had experienced dizzy spells and collapse for 2 years. She did not possess any of the features of the metabolic syndrome [4, 5]. A blood glucose concentration of 2.4 mmol/L had been detected while symptomatic in primary care, and she was referred to the metabolic clinic. There was no family history of diabetes. Direct questioning suggested an association with meals, especially those containing high amounts of carbohydrate. 

**Figure 1:**
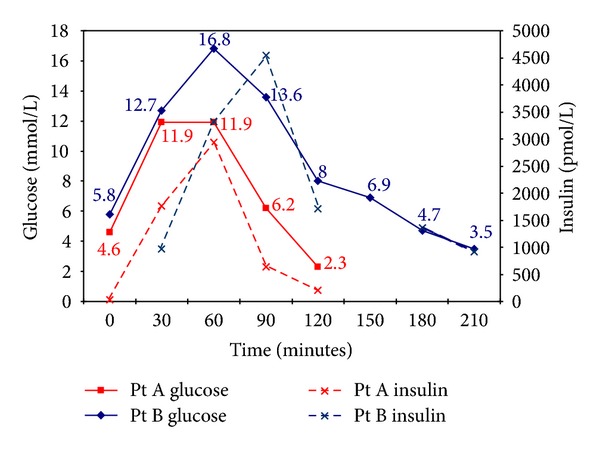
This demonstrates the changes observed in glucose and insulin levelsduring the eGTTs performed on the two patients. It provides a visual understanding of the relationship between blood glucose and insulin levels in the two patients with different presentation patterns.

It was decided to carry out an eGTT. The results of the relevant biochemistry are presented in Table 1(a) and Figure 1. During the test, she became symptomatic (neuroglycopenic symptoms; light headedness, but without clinical evidence of sympathetic counter-regulation; blood pressure and heart rate remained at 117/74 mmHg and 65 bpm, resp.) between 100 and 120 minutes with no evidence of clinical improvement. There did not appear to be any improvement after 130 minutes and the test was abandoned after discussion with the patient. She was given fast acting glucose energy tablets and recovered rapidly while under close medical supervision.

The results were discussed and an association between insulin levels and blood sugar was noted. The pattern observed appeared to fit a reactive pattern, albeit hypoglycaemia occurring early. It was considered that the raised insulin levels prevented the physiological counter-regulation; hence, the patient recovery was compromised. At this point, we decided to repeat the eGTT, but using half the glucose content to establish whether a different clinical, biochemical pattern and recovery took place. Although this modified eGTT was unconventional and may not be useful in determining the diagnosis, it was decided that it might yield important information when compared to the original eGTT. Further, it could be useful to see if smaller glucose load would lead to less severe symptoms. The results of the modified eGTT are presented in Table 1(b). Although the patient once again became symptomatic after 120 minutes she began to recover without medical intervention with the blood sugar increasing to 3.8 mmol/L after 150 minutes. The FFA levels increased in line with the blood sugars suggesting a physiological response to hypoglycaemia. 

### 3.2. Late Hypoglycaemia (Patient B)

Patient B, a 52-year-old man (weight: 118.2 kg; BMI: 38) had experienced severe dizzy spells a few hours following a meal. Although he was not diabetic, he was classified as having the metabolic syndrome (central weight distribution, hypertension, total cholesterol: 4.6 mmol/L; HDL-cholesterol: 0.8 mmol/L; triglycerides: 3.9 mmol/L). Due to the postprandial symptoms an eGTT was carried out and the relevant biochemistry test results are presented in Table 2 and Figure 1. He became symptomatic (neuroglycopenic symptoms: dizziness and blurred vision) after 180 min and the test was stopped at 200 minutes due to lack of recovery, and fast acting glucose energy tablets were given. 

We have presented 2 cases demonstrating differing patterns of postprandial reactive hypoglycaemia. Both patients experienced symptoms due to hypoglycaemia at different times following the glucose load. Patient A did not demonstrate any features of the metabolic syndrome in contrast to patient B. The blood glucose increased rapidly in both patients within 30 minutes; in patient A, it stopped increasing at that point, while in the insulin resistant patient B, it continued to rise. The plasma insulin levels also increased in both patients, albeit the patterns were different as seen in Figure 1. In patient A, plasma insulin concentration increased earlier with peak levels observed after 60 minutes (Table 1(a), Figure 1). This was followed by a rapid decrease in blood glucose. The peak insulin concentration was of smaller magnitude following half the glucose load (Table 1(b)) with improvement in symptoms. In contrast, patient B demonstrated a greater increase in insulin concentration with peak levels occurring after 90 minutes. The decrease in blood glucose also took place later. 

## 4. Discussion

Reactive hypoglycaemia is a phenomenon that may be affected by exaggerated insulin release and insulin resistance [1, 3]. The degree of abnormality of the previous factors may contribute to the different patterns of hypoglycaemia and physiological response as observed in our patients. Insulin levels increased following the glucose load in patient A and was followed by a rapid decrease in glucose levels leading to symptoms. As this patient did not demonstrate the metabolic syndrome, greater insulin sensitivity might have led to the rapid decrease in glucose. The insulin levels although decreasing were perhaps sufficiently high enough during hypoglycaemia to suppress the counterregulatory response which may have resulted in the glucose levels remaining low. The corresponding insulin levels were much lower when the glucose load was halved and may have permitted physiological recovery. Gluconeogenesis as a mechanism of recovery is suggested by the increasing levels of FFA. The improved recovery during eGTT with the halved load of glucose perhaps suggests that patient A might be treated with a diet aimed at reducing the insulin response. Patient B demonstrated fasting insulin levels higher than patient A probably due to underlying insulin resistance. The reduction in glucose was much more gradual, perhaps again due to the action of insulin being blunted by insulin resistance. He suffered from symptoms at a higher glucose concentration compared to Patient A. The insulin levels remained high at this point and may have contributed to the symptoms being prolonged leading to the test being abandoned. 

The primary mechanism in Patient A, who did not demonstrate features of insulin resistance, could have been an exaggerated and rapid increase of insulin release. Rapid absorption of glucose could also have been a factor. Finally, factors influencing insulin and glucagon release, such as incretins and defects in the insulin release mechanism itself, must also be considered. Patient B, in contrast, demonstrated blood glucose and insulin levels during the eGTT more in agreement with insulin resistance. 

Cellular entry of glucose is via the glucose transport (GLUT) proteins which are either insulin-independent or insulin-dependent ones [6–9]. The GLUT 1–3 proteins are ubiquitous and permanently located in the cell surface membrane acting independently of insulin depending instead on glucose concentration. Insulin promotes glucose movement into adipose tissue and skeletal muscle by recruitment of GLUT 4 in these tissues to the plasma membrane [10]. GLUT 2 is the dominant channel in both liver and pancreatic *β* cells. The release of insulin involves alteration in the electrical activity of ß cell ion channels and in ß cell secretory function regulated by soluble *N*-ethylmaleimide-sensitive factor activating protein receptor (SNARE) proteins [11]. Glucose uptake by the ß cells increases cellular ATP production leading to an increased ATP : ADP ratio which in turn closes the ATP sensitive K_ATP_ channels resulting in membrane depolarization [12]. This results in the opening of the voltage gated Ca^2+^ channels leading to fusion of granules containing insulin with the plasma membrane—a step regulated by the SNARE proteins [11]. Glucose-mediated insulin secretion is biphasic consisting of an immediate first phase with limited readily available pool mobilisation followed by a second phase with larger reserve pool utilization [13]. SNARE proteins are involved in both these processes. Released insulin binds to insulin receptor on most tissues of the body resulting in the initiation of the receptor's tyrosine kinase activity, which in turn allows the vesicular stored GLUT 4 channels to be inserted in the cell wall upregulating glucose influx [14–16]. 

A dysfunctional link between insulin and the targeting of GLUT 4 has been shown to contribute to insulin resistance [14, 16]. The pathogenesis of the insulin resistance syndrome is multifactorial. There may be reduced glucose oxidation which could result in impaired insulin secretion and delayed intracellular glucose removal with subsequent delayed cellular entry of glucose and resultant hyperglycaemia. 

Late reactive hypoglycaemias as part of the insulin resistance syndrome may thus be caused by delayed insulin secretion and thus delayed insertion of GLUT 4 [17]. The delayed insertion means that the proportion of glucose entry by insulin-independent GLUT 1–3 has increased thus leaving a smaller proportion for the GLUT 4 to handle. Despite this smaller proportion, the continued hyperinsulinaemia recruits greater numbers of GLUT 4 inappropriately at a later stage where hyperglycaemia is already approaching normoglycaemia and causes rapid entry of glucose into the cells resulting in hypoglycaemia.

Early reactive hypoglycaemia may be the function of an exaggerated incretin effect [3]. The incretin effect explains the phenomenon of oral glucose as a more potent stimulus for insulin release than parenteral glucose of an equal concentration. This is mediated in part by the gut hormones glucagon-like peptide-1 (GLP-1) and glucose-dependent insulinotropic polypeptide (GIP) and could result in excess insulin exocytosis and thus early upregulation of GLUT 4 channels and subsequent hypoglycaemia. In addition to insulin release, GLP-1 suppresses glucagon and thus leaves the patient unable to respond to early hypoglycaemia. Finally, greater oral glucose loads lead to an increased incretin effect which, in turn, may result in more severe reactive hypoglycaemia [18].

There was considerable benefit when both patients understood hypoglycaemia to be the probable reason for their symptoms. However, it was most important that management was tailored to the biochemical pattern observed during the eGTT. It was considered that both patients would benefit from smaller, more regular food intake consisting of carbohydrate of low glycaemic index (GI). Decreased insulin response, as it was hoped, would lead to symptomatic improvement. Currently, both patients have been advised accordingly and are experiencing some benefit.

Similarly, we would consider acarbose as second-line in both patients in order to decrease glucose absorption. We have previously started a patient demonstrating the late hypoglycaemic pattern on sitagliptin (starting at 25 mg and cautiously increasing the dose with patients consent) due to its possibly delaying gastric emptying. We recognise that increased endogenous GLP-1 following a gliptin could lead to even greater insulin levels worsening the symptoms; thus, careful monitoring of this patient was initiated. We would not consider this approach in patients exhibiting early postprandial hypoglycaemia as an exaggerated GLP-1 response which may be causative. We would have some concerns about metformin as it could possibly diminish the physiological response to hypoglycaemia, despite the potential to improve insulin sensitivity. Thus, the mainstay of treatment at present remains focused on reduced glycaemic load and a low GI diet and we will describe this further.

GI is a measure of the effects of carbohydrates on blood sugar levels. GI estimates how much each gram of consumed available carbohydrate (total carbohydrate minus fibre) raises an individual's blood glucose level [19]. Foods with carbohydrates that break down quickly during digestion and release glucose rapidly into the bloodstream tend to be of high GI, while food containing carbohydrates that break down more slowly tend to have a low GI. The concept was developed by Jenkins and colleagues in 1980-1981 at the University of Toronto from their research on the optimal diet for patients with diabetes [20]. Regular consumption of high GI meals, compared with isoenergetic and nutrient controlled low GI meals, results in higher average 24-hour blood glucose and insulin levels, higher C-peptide excretion, and higher HbA1c concentrations in nondiabetic and diabetic individuals [21, 22].

The rate of carbohydrate absorption after a meal, as quantified by GI, has significant effects on postprandial hormonal and metabolic responses. High GI meals produce an initial period of high blood glucose and insulin levels, followed in many individuals by reactive hypoglycaemia [23]. In contrast, a low GI diet may improve the management of diabetes by lowering early postprandial hyperglycaemia and decreasing risk for postabsorptive hypoglycaemia [23]. After a low GI meal, hypoglycaemia and its hormonal sequelae do not occur during the postprandial period owing to continued absorption of nutrients from the gastrointestinal tract and rising hepatic glucose output. Consumption of meals containing identical energy and nutrients can produce markedly different physiological responses [23]. Low GI meals, therefore, would be the meals of choice amongst those with reactive hypoglycaemia.

It has been demonstrated that a low GI meal resulted in significantly lower plasma glucose, serum insulin and plasma GLP-1 than the high GI meal [24]. A further study looked at the short-term effects of a single low or high GI meal on plasma levels of GLP-1, PYY, and insulin in twelve patients [25]. A medium GI dinner was provided, and participants fasted overnight, and in the morning a low or high GI breakfast was consumed and bloods taken every 30 mins for 150 mins. This intervention was repeated with a test meal following a 2-week washout period. There were significantly lower levels of insulin after the low GI breakfast compared to the high GI breakfast [25]. Thus, a low GI diet may be therapeutic for individuals with reactive hypoglycaemia. Further, low GI diets are now recommended in the evidence-based nutrition guidelines for the prevention and management of diabetes (Diabetes U.K. Nutrition Working Group, 2011) [26]. In addition to glycaemic index, glycaemic load also needs consideration. Research has shown that low glycaemic load meals were associated with lower insulin, glucose, and urge to eat ratings but with higher ghrelin [27]. In view of the previous evidence, both low GI and low glucose load diets are the mainstay of patients experiencing reactive hypoglycaemia.

## Figures and Tables

**Table:** (a)

Time (min)	Glucose (mmol/L)	Insulin (pmol/L)	FFA (*μ*mol/L)
0	4.6	32	497
30	11.9	1760	
60	11.9	2940	
90	6.2	640	<50
120	2.3	211	<50
130	2.5		

Blood taken at 0 min showed normal renal, liver, and thyroid biochemistry. Cortisol level was 735 nmol/L.

**Table:** (b)

Time (min)	Glucose (mmol/L)	Insulin (pmol/L)	FFA (*μ*mol/L)
0	4.6	18	727
30	11.9	1170	133
60	9.4	560	<50
90	3.2	134	<50
120	2.2	45	73
150	3.8	16	638

**Table 2:** Biochemistry parameters for patient B following the eGTT with 113 mL of Polycal.

Time (min)	Glucose (mmol/L)	Insulin (pmol/L)	FFA (*μ*mol/L)
0	5.8		
30	12.7	970	650
60	16.8	3320	430
90	13.6	4550	201
120	8.0	1710	119
150	6.9		65
180	4.7	1360	86
210	3.5	920	124
